# Hypoxia Induces Astrocyte-Derived Lipocalin-2 in Ischemic Stroke

**DOI:** 10.3390/ijms20061271

**Published:** 2019-03-13

**Authors:** Fatemeh Ranjbar Taklimie, Natalie Gasterich, Miriam Scheld, Ralf Weiskirchen, Cordian Beyer, Tim Clarner, Adib Zendedel

**Affiliations:** 1 Institute of Neuroanatomy, RWTH Aachen University, 52074 Aachen, Germany; franjbar@ukaachen.de (F.R.T.); ngasterich@ukaachen.de (N.G.); mscheld@ukaachen.de (M.S.); cbeyer@ukaachen.de (C.B.); tclarner@ukaachen.de (T.C.); 2 Institute of Molecular Pathobiochemistry, Experimental Gene Therapy and Clinical Chemistry, RWTH Aachen University Hospital, 52074 Aachen, Germany; rweiskirchen@ukaachen.de

**Keywords:** astrocyte, hypoxia, lipocalin-2, neuroinflammation

## Abstract

Ischemic stroke causes rapid hypoxic damage to the core neural tissue which is followed by graded chronological tissue degeneration in the peri-infarct zone. The latter process is mainly triggered by neuroinflammation, activation of inflammasomes, proinflammatory cytokines, and pyroptosis. Besides microglia, astrocytes play an important role in the fine-tuning of the inflammatory network in the brain. Lipocalin-2 (LCN2) is involved in the control of innate immune responses, regulation of excess iron, and reactive oxygen production. In this study, we analyzed LCN2 expression in hypoxic rat brain tissue after ischemic stroke and in astrocyte cell cultures receiving standardized hypoxic treatment. Whereas no LCN2-positive cells were seen in sham animals, the number of LCN2-positive cells (mainly astrocytes) was significantly increased after stroke. *In vitro* studies with hypoxic cultured astroglia revealed that LCN2 expression is significantly increased after only 2 h, then further increased, followed by a stepwise decline. The expression pattern of several proinflammatory cytokines mainly followed that profile in wild type (WT) but not in cultured LCN2-deficient astrocytes. Our data revealed that astrocytes are an important source of LCN2 in the peri-infarct region under hypoxic conditions. However, we must also stress that brain-intrinsic LCN2 after the initial hypoxia period might come from other sources such as invaded immune cells and peripheral organs via blood circulation. In any case, secreted LCN2 might have an influence on peripheral organ functions and the innate immune system during brain hypoxia.

## 1. Introduction

Ischemic stroke is one of the most common neurological diseases worldwide causing disability and death [1]. Despite the fast restoration of blood and oxygen support of the tissue at risk (reperfusion), managing of the inflammatory cascades is a promising approach to limit secondary damage following tissue reperfusion. Astrocytes are critically involved in the maintenance of cellular functions in the brain under physiological and pathological conditions. They scavenge reactive oxygen species (ROS) and excessive neurotransmitters, control ion and water efflux, release of neurotrophic factors, and control transport mechanisms through the blood–brain barrier (BBB) [2]. Furthermore, astrocytes are part of the regulatory loops of brain intrinsic inflammatory processes and reactive astrocytes are a hallmark of many neuropathological events. Upon activation, they undergo severe morphological and functional changes. The functional phenotype of activated astrocytes can be either detrimental (pro-inflammatory) or protective (anti-inflammatory). Pro-inflammatory astrocytes lose their ability to promote neuronal survival, outgrowth, synaptogenesis, and phagocytosis, and induce the death of neurons and oligodendrocytes [3]. Furthermore, reactive astrocytes may inhibit axon regeneration and neuronal plasticity [4]. In contrast, they are involved in the regulation of water and ion homeostasis, cerebral blood flow, and control of the extracellular level of glutamate, as well as being the source of a variety of neuroprotective factors [5]. Astrocytes can be activated by either innate or adaptive immunity signals. For example, astrocytes get stimulated (directly or indirectly) by lipopolysaccharides (LPS) via Toll-like receptor (TLR)-signaling—a classical innate immunity cascade [6]. Beyond, microglia cells, probably the most important brain resident innate immune cell population, are potent astrocyte activators [7].

After ischemia, peri-infarct astrocytes undergo numerous pathological alterations over time including proliferation, rapid swelling, and increased occurrence of glial fibrillary acidic protein (GFAP) levels in a process termed reactive astrogliosis [8]. These proliferative astrocytes develop the glial scar separating the damaged tissue in the infarct area from the surrounding healthy brain. Furthermore, they modulate adaptive responses of neurons and influence neurological recovery following the initial damage [9]. Thus, astrocytes are essential for the regulation of ischemic tolerance as well as repairing/remodeling neuronal networks by phagocytosis. Under inflammatory conditions, astrocytes secrete Lipocalin-2 (LCN2)—a molecule that has recently been discussed as a valuable biomarker for the prediction of clinical outcome in stroke patients [10]. Besides astrocytes, other types of glial cells and injured neurons also can release LCN2 after ischemia in the reperfusion phase [11]. LCN2, also known as neutrophil gelatinase-associated lipocalin (NGAL), is a secretory glycoprotein belonging to the lipocalin superfamily which binds to its specific cell surface receptors such as 24p3/megalin, thereby modulating several cell functions such as migration, chemokine expression, morphological changes, or apoptosis [12]. In the brain, this molecule may be important for the course of the pathogenesis in stroke, multiple sclerosis, spinal cord injury (SCI), and Alzheimer’s disease (AD) [13]. However, the precise role of LCN2 during the different phases of ischemic stroke, i.e., acute and chronic phase, has not yet been defined.

In the present study, we have studied the time course of peri-infarct LCN2 expression using a well-established rat ischemic stroke model, i.e., transient middle cerebral artery occlusion (tMCAO) model [14]. Since LCN2 is also secreted in a paracrine and endocrine fashion, we have additionally analyzed LCN2 amounts in blood serum of these animals. Further, we attempted to localize LCN2 expression to astrocytes using double-immunofluorescence staining. An *in vitro* mouse hypoxia model [15] with WT and LCN2-knockout (KO) astrocytes was applied to mimic astrocyte responses to ischemic conditions and the effect of LCN2 on inflammatory cytokine expression.

## 2. Results

**Figure 1:**
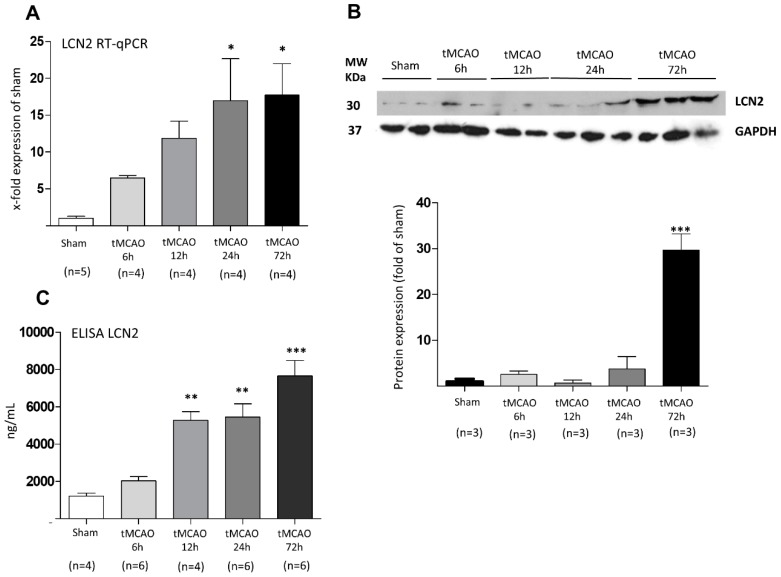
Regulation of LCN2 at (**A**) mRNA and (**B**) protein levels at various time points after tMCAO in the peri-infarct zone of the rat brain. (**A**) mRNA levels increased stepwise and changes became first significant after 24 h. (**B**) LCN2 protein amounts increased in a temporally delayed way as shown in representative WB samples and by densitometric evaluation of immune-labeled bands. (**C**) LCN2 ELISA determinations in the blood serum of rats show a significant increase of LCN2 coinciding well with the above described expression levels in the damaged cortical brain area. * *p* ≤ 0.05, ** *p* ≤ 0.01, and *** *p* ≤ 0.001.

In a first set of experiments, we aimed at investigating whether ischemic stroke induces an increase in LCN2 expression within the peri-infarct cortical brain area. As mentioned in “material and methods”, to avoid contamination of brain tissue with blood, all animals were intracardially perfused with 0.9% NaCl. Real time semi-quantitative PCR (RT-qPCR) was performed on 2,3,5-triphenyltetrazolium-2H-chloride (TTC)-stained tissue isolated at four different time points after stroke onset. Figure 1 shows LCN2 gene expression and protein levels in the peri-infarct zone. We found a stepwise increase of LCN2 mRNA (Figure 1A) in brain tissue with a significant induction at 24 and 72 h post stroke. The Western blot (WB) analysis shows a significant increase of LCN2 protein levels only at 72 h after tMCAO (Figure 1B).

In addition, using an enzyme-linked immunosorbent assay (ELISA), we have measured the amount of LCN2 protein in blood serum samples of the above animals at the same time points. Our data reveal a strong and significant increase of LCN2 serum levels at 12, 24, and 72 h time points. (Figure 1C).

**Figure 2:**
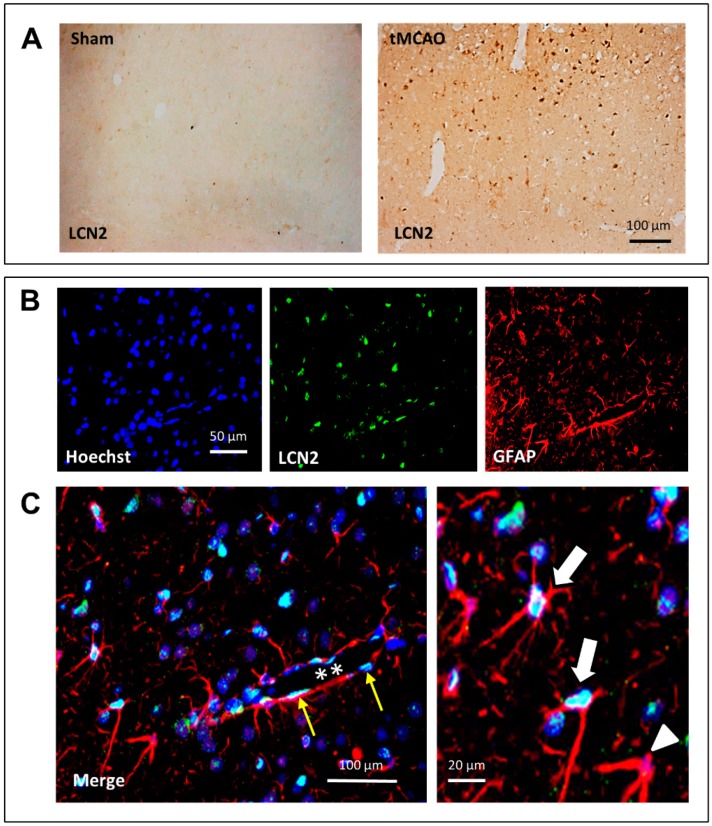
Immunohistochemical (**A**) and double-immunofluorescence (**B**) staining of LCN2 after stroke in the cerebral cortex of tMCAO rats. (A) Numbers of LCN2-positive cells massively increased after tMCAO. In sham-operated animals, almost no LCN2-positive cells were visible. (**C**) Double-labeling revealed that many GFAP-positive (red) astrocytes are also positive for LCN2 (turquoise-white). In (**C**), a blood vessel (stars) crosses the section, and thin elongated endothelial cells (yellow arrows) can be identified. At higher magnification, two LCN2-positive (white arrows) and a LCN2-negative (white arrowhead) can be seen.

In a next step, we performed immunohistochemistry and double-immunofluorescence staining against LCN2 to pinpoint more precisely the cellular source of LCN2 in the peri-infarct area 24 h after stroke onset. As shown in Figure 2A, the number of LCN2-positive cells in respective areas of these rats was massively increased compared to sham-operated animals. In the latter, LCN2 was seen only occasional and at low intensity. Double-immunofluorescence staining revealed that a large portion of LCN2-positive cells within the peri-infarct area represents astrocytes (Figure 2B). In the enlarged merge sections (Figure 2C), most of the GFAP-positive cells also stained for LCN2 (white arrows), whereas only a few GFAP-positive astrocytes were LCN2-negative (white arrowhead). A crossing blood vessel is pictured in Figure 2C (white asterisk). Here, nearby located GFAP-positive astrocytes appear to be LCN2-positive but also flat extended endothelial cells express LCN2 (yellow arrows).

**Figure 3:**
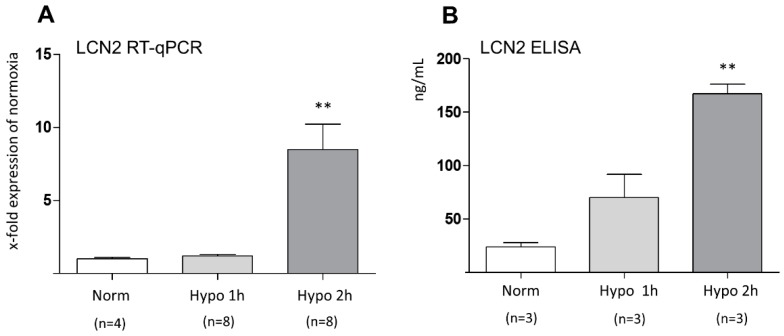
Effect of hypoxia on (**A**) LCN2 gene expression and (**B**) protein levels of LCN2 in cultured primary astrocytes isolated from the cerebral cortex. (**A**) RT-qPCR analysis reveals a significant increase in LCN2 mRNA levels at 2 h post hypoxia. (**B**) LCN2 protein concentrations measured in supernatant of cultured hypoxic astrocytes at 2 h post hypoxia also shows a significant upregulation of LCN2. * *p* ≤ 0.05, ** *p* ≤ 0.01, and *** *p* ≤ 0.001. Norm, normoxia; Hypo, Hypoxia.

**Figure 4:**
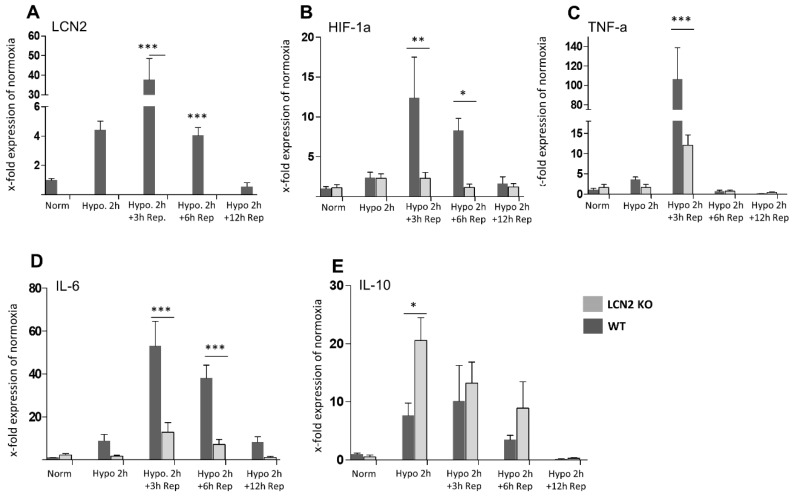
Effect of reoxygenation after an initial 2 h hypoxia on the mRNA expression profiles of LCN2 and various pro- and anti-inflammatory cytokines in mouse astrocyte cultures from the cerebral cortex of WT and LCN2-deficient animals. (**A**) LCN2 expression is significantly but temporarily induced in response in WT astrocytes 3 and 6 h after reoxygenation and returned to control level at 12 h reoxygenation. (**B**–**D**) The profiles of HIF-1α, TNF-α, and IL-6 mRNA expression mainly follow the LCN2 pattern in WT astrocytes. Such an induction of HIF-1α, TNF-α and IL-6 expression was not observed in LCN2-deficient astrocytes. (**E**) In contrast, the anti-inflammatory cytokine IL-10 was significantly elevated in LCN2-deficient astroglia but only moderately elevated in WT glia. Rep, reoxygenation (reperfusion). * *p* ≤ 0.05, ** *p* ≤ 0.01, and *** *p* ≤ 0.001. The number of animals included for reoxygenation was *n* = 5 per time point. Norm, normoxia; Hypo, Hypoxia.

In a second attempt, we analyzed LCN2 mRNA expression in cultured mouse astrocytes from the cerebral cortex with time-limited hypoxia and LCN2 protein levels in the supernatant of these cultures. This setting showed that 2 h after hypoxia onset, both mRNA levels and supernatant LCN2 protein amounts were statistically increased (Figure 3A,B). In a follow-up study, we have investigated the expression levels of several cytokines in WT and LCN2-deficient astrocytes *in vitro* after 2 h of hypoxia. Furthermore, we included reoxygenation groups in the study to more closely mimic the experimental setup applied *in vivo*. First, pro-inflammatory factors such as hypoxia-inducible factor 1-alpha (HIF-1α), tumor necrosis factor (TNF-α), and interleukin-6 (IL-6), were found all induced in hypoxic WT astrocytes, timely coinciding with the LCN2 induction (Figure 4A–D). This stimulatory effect was absent in LCN2-deficient astrocyte cultures (Figure 4B–D). In contrast, we observed that the potent anti-inflammatory cytokine IL-10 which is implicated in limiting host immune responses thereby preventing damage and maintaining normal tissue homeostasis, was only significantly induced in LCN2-deficient but not WT astrocytes (Figure 4E). A further increase of the time period of reoxygenation up to 24 and 48 h did not affect the expression levels of LCN2 and of the other investigated inflammatory marker genes compared to 12 h reoxygenation or basal levels (normoxia). The measured levels after 24 and 48 h almost resembled those after 12 h of reoxygenation and corresponded approximately the basal levels.

## 3. Discussion

Ischemic stroke resulting from focal occlusion of a main artery in the central nervous system (CNS) is considered as one of the most important causes of disability in humans. Initial neuronal tissue damage in the center of undersupply with nutrients and oxygen is followed by delayed metabolic changes and excessive neuroinflammation [16]. In this respect, local inflammatory responses involving brain-intrinsic micro- and astroglia as well as neutrophils, macrophages, and lymphocytes attracted and immigrating through the disrupted BBB are crucial to make a decision between life or death of neurons [17,18]. At an early stage after brain damage, it seems clear that astrocytes and microglia in the vicinity of the injured site are central players: they become activated, change their morphological and functional phenotype and communicate with each other as well as with neurons, endothelial cells and pericytes to form a multicellular “neuroinflammatory network” [19,20,21,22,23]. The cross-talk between the cellular components of this network involves exosomes and cytokine signaling and finally determines the propagation, severity, or termination of local neural inflammatory processes.

In the focus of the present study, we analyzed different aspects of LCN2 expression in this scenario. This protein is a secreted innate immune glycoprotein with a molecular weight of approx. 25 kDa [24]. LCN2 is an acute phase protein that is substantially activated by LPS and the inducible transcription factor nuclear factor kappa-light-chain-enhancer of activated B cells (NF-κB) and HIF-1α [24,25,26]. LCN2 acts as a transport protein, siderophore, modulator of innate immune response, binding partner of gelatinase (MMP-9), and bacterial catecholate-type ferric siderophore, delivering iron to the cytoplasm and functions as an activator/repressor of iron-responsive genes [25,26]. LCN2 generally reveals a ubiquitous increase in expression during inflammatory activity in many organs, e.g., kidney, heart, brain, and liver [27,28,29]. There is emerging evidence that besides its role in peripheral organs, LCN2 is involved in brain injury and the control of neuroinflammation after acute CNS trauma [30] but also in chronic neurodegenerative disorders such as AD [31]. Irrespective of the recently accumulating knowledge about the role of LCN2 in the brain, the exact function has not been fully understood.

In the present study, we have used a well-established rat tMCAO model to mimic the early and prolonged scenario in the brain after a transient ischemic attack [32]. Furthermore, we employed a mouse *in vitro* model using cultured cerebral cortex astrocytes kept under hypoxic conditions [33]. The *in vivo* findings clearly support the view that (i) LCN2 mRNA expression in the peri-infarct area occurs with a short delay of several h after the initial ischemic period and shows a maximum at 72 h post stroke and (ii) is mainly due to astrocytic LCN2 expression. Our view that LCN2 mRNA expression is brain intrinsic is supported by the fact that tissue was perfused with NaCl before sampling, thus making it unlikely that LCN2 mRNA derives from blood cells/vessels. Immunostaining as well as *in vitro* data also pinpoint astroglia as an important source of post-ischemic LCN2 increase and further demonstrate that astroglial LCN2 production/release is required for the subsequent induction of pro-inflammatory cytokine synthesis within the astroglia network. Additionally, LCN2-deficient astrocytes respond with a higher production rate of the anti-inflammatory cytokine IL-10.

These data are in line with previous studies showing an increase of LCN2 levels in astrocytes in response to intracerebral hemorrhage [34], neuroinflammation [35], SCI [36], multiple sclerosis [37], stroke [10,11], and other inflammatory disorders of the brain. Here, we report an induction of LCN2 at mRNA and protein levels within the in peri-infarct area of the brain. Interestingly, the induction of LCN2 mRNA in brain samples increases over time with a maximum at 24 and 72 h. In contrast, WB analysis only revealed faint LCN2 immunolabeled bands which first become stronger after 24 h. This discrepancy between intracerebral mRNA expression and protein levels might be explained by the release of LCN2 into the extracellular space, blood, or cerebrospinal fluid at the time points earlier than 72 h. Secreted LCN2 cannot be detected by WB in NaCl-perfused brain tissue. Our *in vitro* study with cultured astrocytes partially supports this view. Here, we observed a rapid and strong (within 1 h) secretion of LCN2 into the culture supernatant which was absent in LCN2-deficient astrocytes. Another possible scenario could be that astrocytes stop LCN2 secretion after initial release at later time points for unknown reasons. This implies that other sources might account for brain-intrinsic and/or blood-intrinsic LCN2. First, soon after hypoxia the brain experiences a massive blood–brain barrier breakdown at the injured site allowing different immune cells to invade the hypoxic area. This process might be actively stimulated by secreted LCN2. Thus, transformed immune cells within the brain parenchyma may contribute to brain LCN2. This idea is favorable, since it is known that these immune cells synthesize LCN2, too [38]. Both brain-intrinsic and -invaded cells might then contribute to brain and peripheral LCN2. This is supported by the measurement of elevated LCN2 blood levels from 12 h and later. Another possibility is that other sources, i.e., peripheral organs contribute to LCN2 expression. This is a plausible scenario, since signaling from the damaged brain towards peripheral organs is a well-described phenomenon. The increased BBB permeability in stroke allows the passage of mediators such as pro-inflammatory cytokines from brain to periphery which can lead to secondary complications and impair other extra-cranial organs such as spleen [39], heart [40], liver [41], and others. Although cytokines are known to induce LCN2 synthesis [25,42], the identity of signaling molecules that might trigger peripheral LCN2 expression in the context of stroke has yet to be discovered. This aspect is of major importance for the usability of LCN2 as a biomarker to predict disease outcome or severity as suggested by Hochmeister et al. [10]. In stroke, blood levels of LCN2 correlate with disease severity and unfavorable clinical outcome [10,43,44]. In a vascular dementia model, astrocyte-derived LCN2 mediated cognitive impairment and hippocampal damage [45].

In general, LCN2 triggers the shift of astrocytes from an anti- towards a pro-inflammatory phenotype indicated by higher chemokine expression and morphological changes [12]. Therefore, astrocytes seem to be both source and target of LCN2 during brain intrinsic neuroinflammation which affects BBB integrity [46]. Reactive astrocytes might thus be involved in this process by secreting pro-inflammatory factors [47,48]. Cerebral oxygen and nutrient deprivation leads to neuronal damage which is mediated by release of different pro-inflammatory chemokines such as HIF-1α, TNF-α, and IL-6, paralleled by the activation of brain glia cells, platelets, and vascular endothelial cells [49]. LCN2 seems to act as a key regulator of inflammatory processes in brain and attenuates inflammation by upregulation of different pro-inflammatory factors [50]. In our study, together with an increased expression of LCN2 in WT astrocytes, we observed the regulation of pro-inflammatory cytokines such as HIF-1α, TNF-α, and IL-6. This suggests that the pro-inflammatory role of LCN2 is associated with the secretion of inflammatory chemokines. Since LCN2 regulates astrocyte reactivity and is critically involved in BBB breakdown [51], targeting of astrocytic LCN2 might be a promising target to stabilize the BBB and reduce inflammatory processes in the brain. This assumption is strengthened by data from LCN2-deficient mice which revealed lower neurotoxicity after tMCAO [52]. Microglia which usually react faster than astrocytes to pathological stimuli induce astrocyte activation and determine their fate. Similarly, astrocytes not only have the potency to trigger microglial activation, but also to control their cellular functions [53]. One mechanism by which astrocytes trigger inflammatory responses of microglia cells is via the secretion of chemokines such as CXCL10, CCL2, and CXCL12 [54]. These chemokines appear to be under the control of LCN2-signaling [50] and are increased in the chronic stage of ischemia [55].

The exact mechanisms of controlling LCN2 expression and secretion in astrocytes are not known so far. In the context of stroke, both hypoxia itself and the release of danger- associated molecular pattern molecules (DAMPs) following reperfusion are potential candidates to stimulate LCN2 expression. Our *in vitro* results indicate that hypoxia can directly induce the expression of LCN2 in astrocytes. This is in line with studies on tumor cells which were cultured under hypoxic conditions [56]. It has been shown that under hypoxic conditions, LCN2 expression is increased at HIF-1α-positive regions of tumors and parallels HIF-1α expression in those tumor cells. The signaling of LCN2 in astrocytes is not yet understood in detail. However, a recent study showed a possible correlation of HIF-1α and LCN2 expression [45]. It was suggested that HIF-1α increases the expression of LCN2. Our results show a possible positive feedback mechanism of LCN2 on HIF-1α expression and is in support of these published data. However, further experiments are required to confirm this hypothesis. It is evident that HIF-1α expression correlates well with LCN2 expression, thus supporting the fact that HIF-1α is a pivotal control check for LCN2 induction under hypoxia conditions in the brain and during other peripheral organ pathologies. In particular, because HIF-1α represents an early phase expressed factor in response to ischemia [9,10,11,12,13,15]. Our *in vitro* data allow to draw the conclusion that there exists an early HIF-1α/LCN2 interdependent regulation after hypoxia onset in astrocytes. Interestingly, LCN2 and HIF-1α expression decreased stepwise in parallel with no secondary LCN2 induction which could be expected from our *in vivo* data. Here, we found a continuous LCN2 increase until 72 h post stroke. It might be reasonably assumed that the experimental conditions in our *in vitro* approach do not fully model the *in vivo* situation. We used a 2 h hypoxic window *in vitro* with an increasing post-ischemic reperfusion time. Under this specification, we yielded hampered but not dying astrocytes which rapidly recover from their hypoxic status to normal after 12 h. A prolonged reperfusion time *in vitro* up to 24 and 48 h did not change the expression profile of LCN2 and the examined inflammatory markers compared to 12 h and basal levels. One might assume that the initial hypoxic condition was too short. However, we also used longer hypoxic exposure times in preliminary experiments up to 6 h similarly to studies using other glial cell types. Under this regimen, almost all astrocytes were dead or resembled dying cells thus making it impossible to investigate astroglial responses to ischemia. *In vivo*, there might be additional regulators necessary for maintaining LCN2 expression, i.e., NF-*k*B, which appears to account for a biphasic LCN2 regulation. However, the above-mentioned shortcomings of the *in vitro* set-up do not allow, at present, to discriminate between an early and late event in the regulation of LCN2 expression. Another important point is that LCN2 promotes HIF-1α and vascular endothelial growth factor (VEGF) expression in several pancreatic ductal adenocarcinoma cell lines which contributes to an increase in vascularity [57]. The exact mechanism of LCN2-HIF-1α cross-talk is also still unknown.

In summary, our findings broaden the view on the role of LCN2 in the brain during acute injury processes. They place astrocytes in the focus of LCN2-dependent regulation of the “neuroinflammatory network” and highlight the crucial role of astrocytes during the early regulation of neural damage. Astrocytes are the source of LCN2 but also targets for LCN2 [51]. Irrespectively of the initial contribution of astrocytes to brain-intrinsic and secreted LCN2 during the initial hypoxic challenge, additional sources of LCN2 such as invaded leucocytes or peripheral organs have to be taken into consideration to contribute and succeed astroglial LCN2 synthesis. We believe that astrocytes are pacesetters accordingly equipped and able to respond first to pathological challenges, thereby controlling and activating nearby astrocytes/leucocytes. Future studies have to address the additional LCN2 sources after ischemia and their role in the complex brain–body interaction.

## 4. Material and Methods

### 4.1. Animals and Surgery

All experimental procedures and animal care were approved by the Review Board for the Care of Animal Subjects of the district government (LANUV, Recklinghausen, North Rhine-Westphalia, Germany; file reference No. 84-02.04.2013.A212) and are reported in accordance with the ARRIVE guidelines [14,58]. In brief, tMCAO was performed with 12-week-old male Wistar rats (300–350 g) under 2% isoflurane anesthesia (Abbott, Wiesbaden, Germany) and laser Doppler flowmetry (Moor Instruments VMS-LDF2, Axminster, UK) was used to monitor cerebral blood flow (CBF) on the ipsi- and contralateral side during surgery [58]. Animals underwent one hour (h) of tMCAO by use of the intraluminal thread-occlusion technique as reported previously. After exposure of the left common carotid artery (CCA), internal carotid artery (ICA), and external carotid artery (ECA) through a midline neck incision, ECA and CCA were permanently ligated proximally, and the vagal nerve carefully dissected from the ICA. Through the distal CCA a 0.1% poly-L-lysine coated 3-0 monofilament nylon suture (Doccol, Sharon, MA, USA) of 5 cm length was introduced into the ICA and advanced until an immediate drop in baseline CBF occurred. During the entire surgical procedure, the body temperature was maintained at 37–37.5 °C with a heating pad (Fine Science Tools, Heidelberg, Germany). To prevent bleeding the exposed vessels were carefully ligated and a suture was tightened around the filament. One hour after tMCAO and after mask ventilation, brain reperfusion was restored by removal of the filament. Mask ventilation was discontinued, the face mask removed, and animals were breathing ambient air spontaneously thereafter. The neck incision was closed aseptically, and animals were returned to their heated cages breathing spontaneously. Animals underwent neurological and behavioral scoring shortly before euthanasia at the indicated time intervals. Only animals with a solid ipsilateral infarction were included in the study. Brain samples were obtained from ischemic animals at 6, 12, 24, and 72 h post-reperfusion. Sham-operated animals served as controls.

### 4.2. Blood Serum Analysis and Enzyme-Linked Immune Adsorbent Assay (ELISA)

Concentrations of LCN2 in the blood serum and supernatant of cultured astrocytes were measured with an enzyme-linked immunosorbent assay (ELISA) kit following the manufacturer’s protocol (R&D Systems, Minneapolis, MN, USA). For the individual measurements the serum level of LCN2, blood samples were taken from each animal by cardiac puncture at 6 h, 12 h, 24 h, and 72 h after onset of stroke. After centrifugation, serum was immediately snap frozen and stored at −80 °C until analysis. To determine the quantities of *in vitro* secreted LCN2 by astrocytes under hypoxic condition, 1 mL of astrocytic supernatant was obtained and further processed according to the manufacturer’s protocols. According to this instructions (R&D Systems, Minneapolis, MN, USA ), samples were assayed in duplicate and absorbance measured at 650 nm using a microplate reader (Tecan GmbH, Männedorf, Switzerland). Final concentrations were calculated from respective standard curves and expressed as ng/mL.

### 4.3. TTC Staining, RNA Extraction, and Real-Time Quantitative PCR

After ischemia, rats were transcardially perfused with 0.9% NaCl to remove blood from brain vasculature. Brains were rapidly removed and six consecutive coronal sections (2 mm) were prepared and stained in a 2% TTC (Sigma-Aldrich^TM^, Merck, Darmstadt, Germany) solution (15 min, 37 °C). TTC staining is a marker for metabolic function and represents a reliable indicator of ischemic areas after ischemia which can easily be reused for RNA and protein isolation without any substantial loss or degradation [59]. Gene expression studies were performed with tissues isolated from the peri-infarct area or cultured primary astrocytes. Total RNA was extracted using the peqGold RNA TriFast (VWR, Langenfeld, Germany) as previously described [60]. RNA concentration and purity was measured with a NanoDrop 1000 device (PeqLab, Erlangen, Germany). The cDNA synthesis was performed using Moloney Murine Leukemia Virus Reverse Transcriptase (M-MLV) reverse transcription (RT)-kit and random hexanucleotide primer (Invitrogen^TM^, Thermo Fisher Scientific, Langerwehe, Germany) adjusting the concentration to 1 µg/mL of total RNA. RT-qPCR analysis was performed using the MyIQ detection system (Biorad, München, Germany). Relative quantification was calculated by the ΔΔCt-method using the qbase+software (Biogazelle, Gent, Belgium). Data were expressed as relative amount of the target gene to the amount of a reference gene (CycloA). The values of sham animals (*in vivo*) or normoxia (*in vitro*) were set to 100%. Data of interest are given as relative expression. A list of used primers and analyzed genes is given in Table 1.

### 4.4. Immunohistochemistry and Immunofluorescence Double-Labeling

For immunohistochemistry, the brains were fixed by transcardial perfusion with 4% paraformaldehyde (pH 7.4). Subsequently, the brains were placed in the same fixative overnight. Thereafter, tissue specimens were embedded in paraffin (Merck, Darmstadt, Germany) and sectioned in 5 µm thickness. The paraffin-sections were deparaffinized and treated for heat-induced epitope retrieval (HIER) using Tris/EDTA buffer (pH 9, 20 min). Then, slices were exposed to 10% goat serum for blocking of nonspecific binding (Sigma-Aldrich TM, Merck, Darmstadt, Germany) for 60 min and afterwards incubated overnight at 4 °C with the anti-LCN2 antibodies (for details of antisera see Table 2). The next day, slices were washed and treated with H_2_O_2_/PBS (0.3%, 30 min) (Roth, Karlsruhe, Germany) to block endogenous peroxidase. Afterwards, sections were exposed to the appropriate biotinylated secondary antibody (1:50, Vector Laboratories, Burlingame, CA, USA). Finally immunoreaction was visualized using 3,3′-Diaminobenzidine (DAB) as substrate (DAKO, Carpinteria, CA, USA). For immunofluorescence studies, sections were processed as described above and appropriate secondary antibodies conjugated with fluorescent dyes Alexa 488 or 594 (1:500; Invitrogen^TM^, Thermo Fisher Scientific, Langerwehe, Germany) were applied. Finally, cell nuclei were counterstained with Hoechst 33,342 (Invitrogen^TM^, Thermo Fisher Scientific, Langerwehe, Germany). Fluorescence images for qualitative expression analysis were acquired with a Leica DMI 6000 B microscope (Leica Biosystems, Nussloch, Germany). A list of the used antibodies is shown in Table 2.

### 4.5. SDS-PAGE and Western Blot Analysis

Frozen brain samples were homogenized in RIPA buffer consisting of 150 mM NaCl, 1% (*v*/*v*) Nonidet P-40 (Igepal, Sigma-Aldrich TM, Merck, Darmstadt, Germany), 0.1% SDS (sodium dodecyl sulfate), 0.5% sodium deoxycholate, and 50 mM Tris-HCl, pH 8.0 supplemented with a protease inhibitor cocktail (Complete Mini, Roche Diagnostics, Mannheim, Germany). Protein concentrations were determined using the PierceTM BCA Protein Assay kit (Thermo Fisher Scientific, Waltham, MA, USA) according to the manufacturer’s protocol. Protein samples (25 µg per lane) were separated by 8 to 12% (*v*/*v*) discontinuous sodium dodecyl sulfate-polyacrylamide gel electrophoresis (SDS-PAGE) and transferred to a polyvinylidene difluoride (PVDF) membrane (Roche Diagnostics, Mannheim, Germany). After blocking with 5% skimmed milk for 1 h at room temperature, PVDF membranes were incubated with anti-LCN2 antibody (Table 2) overnight at 4 °C. After washing with 0.15 M NaCl 50 mM Tris-HCL, 0.05% Tween 20 (TBS-T), membranes were incubated with the appropriate horseradish peroxidase-conjugated secondary antibody for 2 h at room temperature. Visualization was achieved with the enhanced chemoluminescence method (ECL Plus; Thermo Fisher Scientific, Waltham, MA, USA) using a standard protocol. Glyceraldehyde 3-phosphate dehydrogenase (GAPDH) was used as a loading control. Densitometric evaluation was performed using ImageJ software (Free Java software provided by the National Institutes of Health, Bethesda, Maryland, USA).

### 4.6. Primary Astrocyte Cultures from LCN2-Deficient and Wild Type Mice

*LCN2 deficient* mice were kindly provided by the laboratory of Mak and colleagues. Details about the mice strain are given elsewhere [61]. Briefly, a targeted mutation has been introduced to replace most of the LCN2 coding region, including exons 1–5, with the PGK-neo cassette, thus leading to a functional knock out in all tissues including the CNS. For more information, please see the original reference [61]. Primary astrocyte cultures were prepared from the cerebral cortex of early postnatal WT or LCN2-deficient Bl6 mice at day 3, as described previously [62]. In order to obtain cell cultures from both genotypes (WT and LCN2^-/-^), single pup cultures were prepared from transgenic and non-transgenic WT littermates. Briefly, after dissecting the brain and removing the meninges, tissues were incubated in ice-cold HEPES buffer. The cortices were transferred into 10 mL of ice-cold Dulbecco’s Modified Eagle’s Medium (DMEM, Gibco TM, Thermo Fisher Scientific, Waltham, MA, USA) supplemented with 10% fetal calf serum, 50 U/mL penicillin, and 50 μg/mL streptomycin (DMEM 10%), and then homogenized mechanically with a 10 mL and 1 mL pipette. The cell suspension was strained through a 70 µm cell strainer, in order to produce a single cell suspension. Cells were pelleted by centrifugation at 800 × g for 5 min, the supernatant discarded and 10 mL of DMEM 10%, pre-warmed to 37 °C, was added. The suspension was transferred to Poly-L-Ornithine (PLO)-coated flasks and incubated at 37 °C and 5% CO_2_. Astrocytes were allowed to attach and grow undisturbed for 4 days (d). Afterwards, other glial cell types were removed from the culture by shaking the flasks at 37 °C for 2 h at 120 rpm and replacing the medium with fresh DMEM 10%. Medium was changed every 2 to 3 d until the astrocyte layer was confluent. To passage the astrocyte culture, medium was discarded and astrocytes were detached using 0.1% trypsin in 2% EDTA/PBS. Cells were collected in DMEM 10%, centrifuged at 300 × g and room temperature for 5 min and suspended in fresh DMEM 10%. Each flask was passaged 1:3 into new PLO-coated flasks with a total volume of 10 mL Astrocytes were used for experiments in passage 3. The WT C57BL/6J mice were obtained from the Jackson Laboratory (Jackson Laboratory, Bar Harbor, ME, USA). All animals used in the present study were acquired and cared for in accordance with the Federation of European Laboratory Animal Science Associations recommendations.

### 4.7. In Vitro Hypoxia

As described previously [19], a self-constructed cube-shaped hypoxia chamber was streamed with inert nitrogen (N_2_) gas to replace aerial oxygen (O_2_) 30 min prior to hypoxia incubation. The O_2_ concentrations were routinely measured simultaneously in the hypoxia chamber and in supernatant of selected culture wells using two separate O_2_ measurement probes. In the hypoxia chamber, O_2_ concentrations very rapidly in the range of few min decreased to values of 0.1% after floating with N_2_ and then remained on this level throughout the hypoxia period. In the cell supernatant, we observed a rapid decline of O_2_ concentrations within several min reaching levels of < 0.5% which, again, remained stable throughout N_2_ floating.

**Figure 5:**
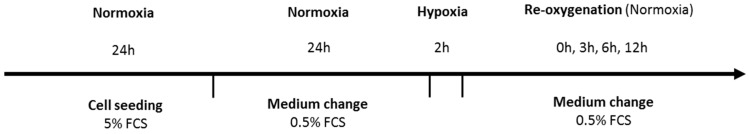
Schematic illustration of the *in vitro* hypoxia model used in this study.

Cells were exposed to hypoxic conditions lasting for 2 h and subsequently processed for analysis or subjected to oxygen reperfusion and kept for another 3, 6, and 12 h with starving medium (FCS 0.5%, (*v*/*v*) pen/strep 0.5%). A schematic illustration of the treatment protocol is shown in Figure 5. The 2 h hypoxia window was applied, since preliminary studies have shown that this duration of hypoxia is sufficient to create a large fraction of hypoxic and hampered astrocytes *in vitro* without killing these cells within the scheduled experimental setting. In contrast, increasing hypoxia duration up to 6 h yielded a large number of dead or dying astrocytes thus making it impossible to investigate O_2_ deprivation in relation to inflammatory responses. Similar observations were made in previously published studies using other brain glia cell types [16].

### 4.8. Data Analysis

The numbers (*n*) of animals in the individual groups used in our study experiments are mentioned in the figures. A total of five independent *in vitro* experiments were performed with each 5 wells per group. The data are presented as arithmetic means ± SEM. Statistical differences between groups were analyzed by one-way analysis of variance (ANOVA) followed by Tukey´s post-hoc test by using GraphPad Prism 5 (GraphPad Software, San Diego, CA, USA). The statistical differences between WT and knock out (KO) groups (*in vitro*) were performed by using two-way ANOVA method. Differences between groups were considered statistical different at a *p*-value of *p* ≤ 0.05. The *p*-values are given as * *p* ≤ 0.05, ** *p* ≤ 0.01, and *** *p* ≤ 0.001.

## Figures and Tables

**Table 1:** List of primers used in the study.

Primer	Sequence	Product Size (bp)	AT °C
LCN2 (mouse)	5′ GCAGGTGGTACGTTGTGGG 3′ 3′ CTCTTGTAGCTCATAGATGGTGC 5′	95	65
LCN2 (rat)	5′ CAGAACTTGATCCCTGCCCC 3′ 3′ GCTGTACATGGTAAAGCGGC 5′	147	59
HIF-1α	5′ TCAAGTCAGCAACGTGGAAG 3′ 3′ TATCGAGGCTGTGTCGACTG 5′	198	65
TNF-α	5′ ACCCCTTTACTCTGACCCC 3′ 3′ GAGTCCTTGATGGTGGTGC 5′	189	62
IL-6	5′ GATACCACTCCCAACAGACCTG 3′ 3′ GGTACTCCAGAAGACCAGAGGA 5′	223	64
IL-10	5′ GCTCTTGCACTACCAAAGCC 3′ 3′ CTGCTGATCCTCATGCCAGT 5′	112	65
CycloA (rat)	5′ GGCAAATGCTGGACCAAACAC 3′ 3′ TTAGAGTTGTCCACAGTCGGAGAT 5′G	196	65
CycloA (mouse)	5′ TTGGGTCCAGGAATGGCAAGA 3′ 3′ ACATTGCGGGAGCAGATGGGGT 5′	148	64

AT = annealing temperature, bp = base pairs.

**Table 2:** List of antibodies.

Antibody	Company	WB	IHC	IF
LCN2	Santa Cruz Biotechnology, Santa Cruz, CA, USA	1:1000	1:500	1:300
GFAP	Bioss, Woburn, MA, USA	---------	----------	1:300
GAPDH	Sigma-Aldrich^TM^, Merck, Darmstadt, Germany	1:4000	----------	------
Goat-anti-rabbit (488)	Invitrogen TM, Thermo Fisher Scientific, Langerwehe, Germany	----------	----------	1:500
Goat-anti-chicken (594)	Invitrogen TM, Thermo Fisher Scientific, Langerwehe, Germany	----------	----------	1:500
Goat-anti-rabbit	Vector Laboratories, Burlingame, CA, USA	----------	1:50	------

WB = Western blot; IHC = immunohistochemistry; IF = immunofluorescence.
